# The Influence of Micronutrient Trace Metals on *Microcystis aeruginosa* Growth and Toxin Production

**DOI:** 10.3390/toxins14110812

**Published:** 2022-11-21

**Authors:** Jordan A. Facey, Jake P. Violi, Josh J. King, Chowdhury Sarowar, Simon C. Apte, Simon M. Mitrovic

**Affiliations:** 1 School of Life Sciences, University of Technology Sydney, Ultimo, NSW 2000, Australia; 2 CSIRO Land and Water, Lucas Heights, Sydney, NSW 2234, Australia; 3 Prince of Wales Clinical School, University of New South Wales, Kensington, NSW 2052, Australia

**Keywords:** cyanobacteria, microcystin, growth limitation

## Abstract

*Microcystis aeruginosa* is a widespread cyanobacteria capable of producing hepatotoxic microcystins. Understanding the environmental factors that influence its growth and toxin production is essential to managing the negative effects on freshwater systems. Some micronutrients are important cofactors in cyanobacterial proteins and can influence cyanobacterial growth when availability is limited. However, micronutrient requirements are often species specific, and can be influenced by substitution between metals or by luxury uptake. In this study, *M. aeruginosa* was grown in modified growth media that individually excluded some micronutrients (cobalt, copper, iron, manganese, molybdenum) to assess the effect on growth, toxin production, cell morphology and iron accumulation. *M. aeruginosa* growth was limited when iron, cobalt and manganese were excluded from the growth media, whereas the exclusion of copper and molybdenum had no effect on growth. Intracellular microcystin-LR concentrations were variable and were at times elevated in treatments undergoing growth limitation by cobalt. Intracellular iron was notably higher in treatments grown in cobalt-deplete media compared to other treatments possibly due to inhibition or competition for transporters, or due to irons role in detoxifying reactive oxygen species (ROS).

## 1. Introduction

Cyanobacterial blooms are common in freshwater systems and threaten anthropogenically and environmentally important resources [1]. A primary driver of cyanobacterial blooms is high nutrient concentrations, or eutrophication [2]. While the link between phosphorous and nitrogen and cyanobacterial growth is well established [3,4,5,6,7,8], there are instances where seemingly favourable conditions do not instigate a bloom, suggesting the importance of additional factors. There is growing evidence that micronutrients can regulate cyanobacterial growth and can act as a limiting factor [5,9,10,11,12]. Up to a third of all microbe proteins contain a metal cofactor [13], and as such, trace metals are clearly vital to maintaining cellular functions. In cyanobacteria, metals play a variety of roles, but are most often associated with photosynthetic electron transport in the thylakoids and the assimilation of macronutrients nitrogen and phosphorus [14,15,16,17]. Their capacity to limit cyanobacterial growth has been demonstrated by in situ nutrient enrichment bioassays [10,11,18,19,20,21] and culture studies [12,22,23,24,25,26,27]. Given the importance of trace metals as micronutrients as well as their toxicity at high concentrations [9], maintaining a balance in intracellular trace metal quotas is essential for optimal cellular metabolism (for a comprehensive review of trace metal uptake and transport pathways see [15]). However, little is known about how cyanobacteria respond to extended periods of low metal availability. 

*Microcystis aeruginosa* is among the most common and widely distributed bloom-forming cyanobacterial species found in freshwater systems [28,29,30]. *M. aeruginosa* can produce hepatotoxic microcystins, a group of cyclic heptapeptides with >279 isomers of varying toxicities [31,32,33,34]. Microcystins predominantly accumulate in the liver, but also in the kidney, heart, gonad and brain of animals [35,36]. They inhibit protein phosphatases PP1 and PP2A, damage membrane integrity, promote oxidative stress and cause tumors [31,37,38,39]. The environmental conditions conducive to increased cyanobacterial toxin production are still widely debated. There is some evidence that the rate of microcystin production is linked with various physical and chemical factors, for example macronutrients [40], light [41,42], pH and temperature [33,42]. While other studies indicate that toxin production is simply related to cell division and growth [43,44]. Neilan et al. [33] reasoned that while there is a strong correlation between microcystin production and growth rate, a more complex relationship with certain nutrients and physiochemical conditions exists. An early study by Lukac and Aegerter [22] observed that microcystin production was stimulated in response to suboptimal iron availability and suggested microcystin may assist in the acquisition of metal ions. Since then, numerous studies have examined the relationship between microcystin production and iron, some of which support the findings of Lukac and Aegerter [22], for example Alexova et al. [45], Yeung et al. [46] and Sevilla et al. [47]. While others found a positive relationship between iron concentration and microcystin production [23,48,49]. Other trace metals have received significantly less attention, or in some cases none [17]. 

Identifying the environmental conditions driving the increase in cyanobacterial blooms and stimulating cyanotoxin production is essential to developing effective management strategies aimed at protecting the ecological and economic value of freshwater systems. The role of micronutrients in *M. aeruginosa* bloom formation and toxicity has received little attention, except for iron. In this study, we aim to determine the importance of certain micronutrients (iron, cobalt, copper, manganese and molybdenum) for the optimal growth of *M. aeruginosa* by excluding them from culture media. Specifically, we aim to determine the effect of low levels of various micronutrients on (1) *M. aeruginosa* growth rate (2) cell volume (3) intracellular accumulation of iron and (4) production of the cyanotoxin microcystin-LR. 

## 2. Results

**Figure 1:**
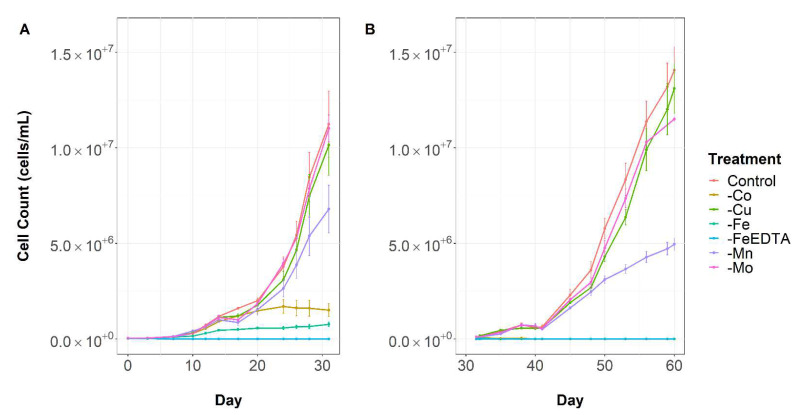
*Microcystis aeruginosa* growth through time under variable micronutrient conditions. (**A**) Transfer 1 and (**B**) Transfer 2. Error bars are standard error of the mean.

**Figure 2:**
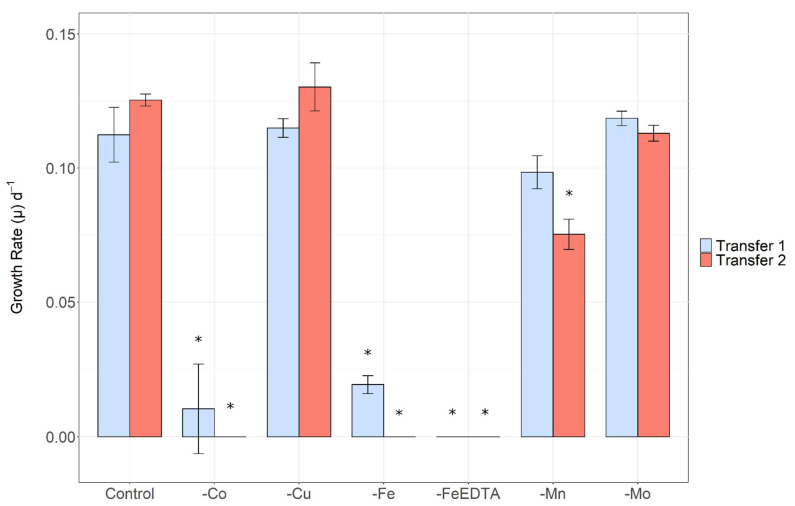
Specific growth rate in treatments exposed to depletion of different micronutrients across two transfers. Asterisk (*) denotes significant difference relative to the control of the same transfer (One-way ANOVA: *p*-value < 0.05).

There were notable decreases in the growth of *Microcystis aeruginosa* when depleted of iron, cobalt and manganese (Figure 1). Iron starved cultures demonstrated the most severe signs of growth limitation, becoming limited after 12 days of growth. Without the presence of both iron and EDTA, *M. aeruginosa* growth reduced further and was negligable. Cobalt deplete cultures showed signs of growth limitation after 24 days and manganese after 30 days. There were no significant differences in growth between treatments starved of copper or molybdenum compared to the control. Similar trends were observed in specific growth rate (Figure 2). There were no significant differences observed between the control, copper and molybdenum. Iron and cobalt depletion both caused significant reductions in specific growth rate. Manganese depletion had a minor effect on growth rate in the first transfer, however after this, growth rate was significantly decreased compared to the control.

**Figure 3:**
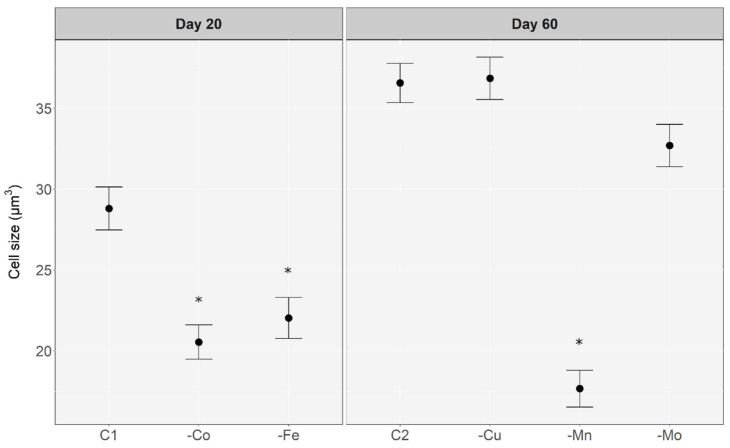
Scatterplot of cell volume relative to cells in the control treatment. Cell volume was measured once the treatment exhibited a growth limitation and compared to the control cell volume at the same time point (C1—control day 20; C2—control day 60). Data for the FeEDTA treatment was not obtained due to the rapid decrease in cell density. Asterisk (*) denotes significant difference relative to the control. Error bars are ± standard error of the mean.

There were significant differences between *Microcystis aeruginosa* cell volume when exposed to different micronutrient conditions (Kruskal–Wallis ANOVA, *p*-value < 0.01). Figure 3 illustrates changes in cell volume under different treatments. Cell volume was measured when treatments began showing decreased growth compared to the control, or otherwise at the completion of the experiment. Treatments that exhibited signs of growth limitation were significantly smaller than control cells. Iron and cobalt depletion caused a ~25% reduction in cell volume (***μ*** = 22.03 ± 1.27 μm and ***μ*** = 20.54 ± 1.06 μm, respectively). Manganese deprivation appeared to have a large effect on cell volume, which decreased ~52% compared to the control (***μ*** = 17.66 ± 1.14 μm).

**Figure 4:**
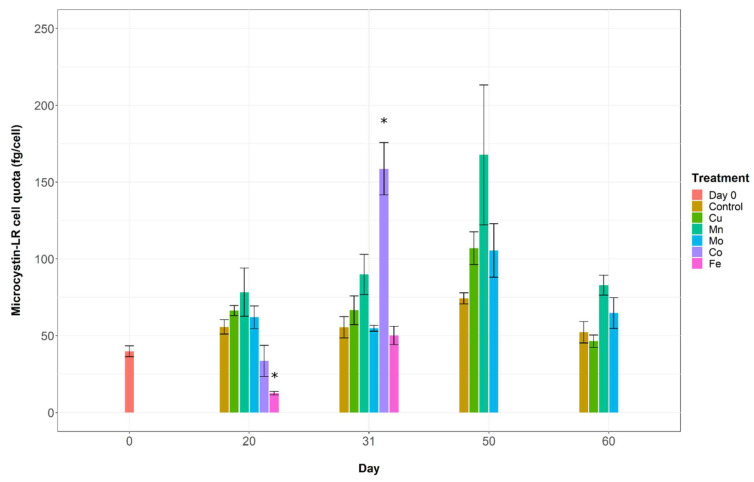
Changes in intracellular microcystin-LR cell quotas throughout the experiment. Samples from the FeEDTA treatment had insufficient sample mass for analysis so are excluded. Error bars are standard error of the mean. Asterisks (*) denote significant difference to control at same time point (PERMANOVA: *p*-value < 0.05).

Intracellular microcystin-LR (MC-LR) concentrations fluctuated through time and with treatment (Figure 4). The concentration of intracellular MC-LR in the -Fe treatment was significantly lower than the control after 20 days (PERMANOVA: *p*-value 0.002) whereas in the -Co treatment microcystin-LR concentration was significantly higher than the control after 31 days (PERMANOVA: *p*-value 0.014). The decreased intracellular microcystin-LR concentration in the -Fe and the elevated concentration in the -Co treatment both corresponded with notable limitation of growth in their respective treatments. There were no other statistically significant differences between treatments and the control at any time points. 

**Figure 5:**
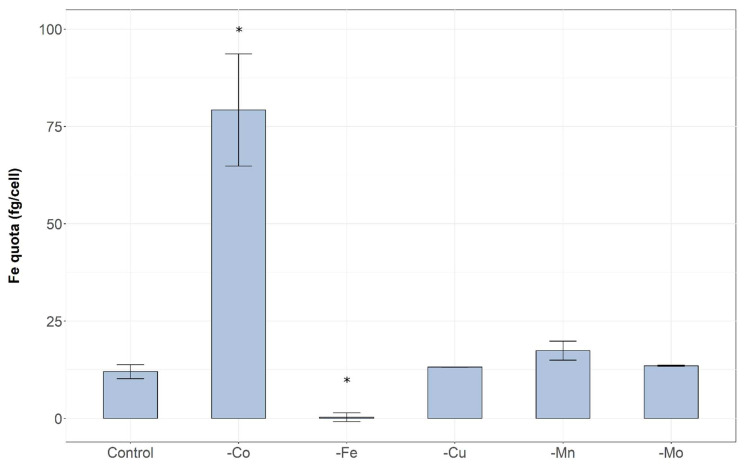
Differences in the intracellular quota of iron in treatments depleted of different micronutrients after 31 days. Samples from the FeEDTA treatment had insufficient sample mass for analysis so are excluded. Error bars are standard error of the mean. Asterisk (*) denotes significant difference compared to the control (One-way ANOVA: *p*-value < 0.05). A log10 transformation was performed to satisfy the assumptions of parametric statistical analyses.

The intracellular iron quota in the treatment starved of cobalt was much higher than the control and all other treatments after 31 days of growth (One-way ANOVA: *p*-value 0.001) (Figure 5). The iron deplete treatment exhibited significantly lower intracellular iron concentration compared to all other treatments. There were no significant differences between treatments -Cu, -Mn and -Mo treatments compared to each other or to the control.

## 3. Discussion

*Microcystis aeruginosa* was grown in batch cultures under different trace metal conditions to assess how micronutrient deprivation affects growth, cell morphology, toxin production and iron regulation. The removal of several trace metals had demonstrable effects on growth (Figure 1), confirming they are required by *Microcystis aeruginosa* for optimal cellular functioning. The growth of *M. aeruginosa* in cultures depleted of iron, cobalt and manganese exhibited decreased maximum cell density and growth rate compared to the control treatment grown in trace metal replete conditions. As hypothesised, iron limitation induced the most pronounced effects, with severe limitation of growth observable after 12 days of exposure to iron-deplete conditions. This result is not surprising given the wide range of iron-requiring functions in cyanobacteria, for example, iron is required as a cofactor of many enzymes, detoxifies reactive oxygen species (ROS) and has a direct role in electron transport [5,14,23,45]. Further, growth limitation of cyanobacteria by iron has previously been observed in both culture studies [22,23,25,46,50] and field conditions [19,21]. Chlorosis was evident within 12 days of iron starvation, perhaps indicating nitrogen colimitation, as suggested by Sherman and Sherman [51]. This is likely due to the role of iron in nitrate reduction and assimilation [52].

Due to the low solubility of iron in oxygenated, circumneutral waters [53], a chelator or ligand, such as ethylenediaminetetraacetic acid (EDTA), is often added to increase iron’s bioavailability in culture experiments [54]. Given that many cultured cyanobacterial genera are effectively grown in media containing chelating agents it is apparent that they are capable of utilizing chelated iron, however unchelated inorganic iron is often reported as the preferred form for phytoplankton and the most bioavailable [55]. Chelated iron still plays an important role in cyanobacterial growth, as observed by Lange [56] who noted that the addition of chelated iron regularly enhanced growth of culture grown cyanobacteria. Uptake of this form of iron relies on reductive or siderophore-assisted pathways [55]. In the present study, the absence of the chelator EDTA along with iron (Treatment E) had a large negative impact on *M. aeruginosa* growth. Growth was negligible following day 0 and was significantly less than eliminating iron alone (Treatment D). In Treatment D, EDTA may have chelated some trace levels of iron contamination in the day 0 solution, causing the small degree of growth observed. By day 10 iron concentration was below detection limit in Treatment D (Supplementary Data, Table S1) and growth rapidly plateaued.

Cobalt deficiency also had a large negative effect on the growth of *M. aeruginosa*, consistent with a previous study [12] that found *M. aeruginosa* required cobalt in a concentration of ~0.06 μg/L to sustain optimal growth. The importance of cobalt was also noted by Downs et al. [10] who observed an increase in primary productivity upon addition of cobalt during a bloom of *Anabaena flos-aquae*. Further, some marine cyanobacteria (e.g., *Prochlorococcus*, *Trichodesmium* and *Synechococcus*) appear to have an absolute cobalt requirement [57,58,59]. Cobalt is predominantly linked with cobalamin (vitamin B_12_), required for transfer of methyl groups and rearrangement reactions in cellular metabolism [13,59,60]. Cyanocobalamin was present in the culture media for all treatments as it is a part of the vitamin mix in MLA media [54]. Growth was suppressed despite the presence of cobalamin in the media, suggesting that *M. aeruginosa* lacks the appropriate transporters to acquire cobalamin from its surroundings and therefore the cyanocobalamin added in MLA media is not bioavailable to *M. aeruginosa*. The cobalt associated with cyanocobalamin likely cannot be utilised for other means. These results may indicate that cobalamin requirements differ between cyanobacterial species. This is supported by Helliwell et al. [60] who found that two strains of *Microcystis*, along with the vast majority of cyanobacteria, lack the full suite of genes required for the synthesis of cobalamin. Instead, many genera synthesise pseudocobalamin, a structural variation of the form added to MLA media. Cells exposed to cobalt depletion may have been growth limited due to the inability to synthesise pseudocobalamin combined with the non-bioavailability of cyanocobalamin. Alternatively, Rodriguez and Ho [59] conducted batch culture experiments using *Trichodesmium* with varying concentrations of Co and cobalamin. Like the present study, low cobalt concentrations appeared to limit *Trichodesmium* growth. Upon addition of cobalamin, growth was elevated, indicating *Trichodesmium* can utilise cobalamin and acquire its biological demand for cobalamin from the surrounding media. However, cultures used by Rodriguez and Ho [59] were not axenic, therefore other bacteria may have influenced Co dynamics. 

As expected, in the iron deplete treatment intracellular iron quota was negligible due to the exclusion of iron from the growth medium. Interestingly, intracellular iron quotas after 31 days were much higher in the cobalt deplete treatment compared to the control and all other treatments (Figure 5). This expands upon the findings of our previous study [12] in which a similar negative relationship between intracellular iron and cobalt availability was observed. In our earlier study cobalt alone was tested, the present study builds upon these findings as we demonstrate that this relationship was not observed with any other micronutrients tested. In higher plants high cobalt concentrations induce Fe deficiency by reducing absorption and inhibiting transport [61,62,63]. This may indicate that cyanobacterial Co transporters also bind to Fe. When there are low Co concentrations more of these binding sites may be used for Fe or, alternatively, more transporters may be produced in response to low Co which can then be utilised by Fe. We can also speculate that iron was selectively transported into cells undergoing cobalt deficiency due to its role in the detoxification of ROS [64] that may have been produced due to the lack of cobalt in the growth media. However, ROS were not measured in this experiment. Further, the manganese deplete treatment was also showing signs of growth limitation after 31 days but the increase in iron quota was not apparent in this treatment, reinforcing that this relationship appears specific to cobalt. In future studies it may be valuable to focus upon Fe kinetics during periods of cobalt limitation to understand this relationship. 

Manganese plays a crucial role in photosynthesis and growth. In cyanobacteria, Mn plays a similar role to iron, as it is a crucial component of PSII, where four Mn atoms form the core of the water-splitting site. It may also scavenge and detoxify ROS [65]. Consistent with other studies [66,67], iron limitation has a more severe effect on phytoplankton growth compared to manganese due to its induction of extensive protein degradation in both PSII and PSI. This is illustrated by the relatively long period taken for manganese limitation to become apparent compared to iron (Figure 1). 

As expected, molybdenum had no effect on *Microcystis aeruginosa* growth rate. Molybdenum is important to heterocystous cyanobacteria for the assimilation of inorganic nitrogen [68,69] and does not appear to be required by the non-heterocystous *M. aeruginosa*. More surprisingly, copper deficiency did not limit *M. aeruginosa* growth over 60 days. Copper is reportedly necessary for phytoplankton growth given that it is a component of the thylakoid membrane [15,16], as well as cytochrome oxidase and plastocyanin in the electron-transport chain [14,70]. However, Sunda [71] reinforces that cellular trace metal concentrations and requirements differ among phytoplankton species. Further, some copper-containing proteins (such as plastocyanin and Cu/Zn-SOD) are readily substituted for iron-containing proteins (cytochrome *c_6_* and Fe-SOD) [71]. These substitutions may reduce the copper requirement of *M. aeruginosa* and prolong the period within which an intracellular copper store can sustain cellular functioning and optimal growth in copper-depleted conditions. 

Treatments undergoing a growth limitation (-Co, -Fe, -Mn) had a significantly smaller cell volume than control cells (ANOVA: *p*-value < 0.05) (Figure 3). Whereas there were no differences between the cell volume of the control treatment and treatments not showing growth limitation (-Cu, -Mo). Given that all treatments undergoing growth limitation exhibited a decrease in cell volume relative to the control, it is likely a general morphological response to an environmental stressor. This has been previously observed by Yeung et al. [46], who found that iron limitation caused a decrease in cell size of *Microcystis aeruginosa* grown in continuous culture. The reduction in cell volume may be part of a reversable downregulation of physiological rates where cell growth and metabolism are decreased in response to stress caused by micronutrient deficiency [72]. A similar process has been observed in cyanobacteria during nitrogen starvation in which a dormant, chlorotic state is established until favourable nutrient conditions are attained [73]. 

It has previously been proposed that microcystins function as a siderophore, a biogenic ligand that assists in the acquisition of iron by facilitating their transport across the cell membrane [22,49,74]. Siderophores increase iron bioavailability via a reduction of the less-bioavailable ferric iron (Fe^3+^) to form ferrous iron (Fe^2+^) [4,45,75,76]. However, Klein et al. [77] showed that Fe^3+^ forms weaker complexes with microcystin-LR than is typical of other siderophores, and proposed that microcystins are more likely to regulate iron via intracellular processes or by acting as a shuttle across the cell membrane. Recent studies suggest that nutrient acquisition systems like siderophores may exist for other metallic nutrients [74]. Microcystins do form complexes with some metal ions besides iron, such as Zn^2+^, Cu^2+^, and Mg^2+^ [78,79], however these have yet to be thoroughly studied in relation to microcystin-LR production. Our results show no stimulation of microcystin-LR production upon iron limitation, as would be expected if it was functioning as an iron-scavenging siderophore released under stress. This is consistent with findings by Li et al. [23] and Amé and Wunderlin [48]. Similarly, intracellular microcystin-LR was not significantly higher than the control in -Cu, -Mo, or -Mn at any time points. In the -Co treatment, microcystin-LR was significantly higher than the control on day 31, when growth limitation was most severe. If a more generalised relationship existed—for example stimulated in response to oxidative stress, microcystin-LR concentration would likely have also been increased by iron and manganese limitation. The relationship between cobalt and microcystin-LR production has not been examined in depth, however these results may provide preliminary evidence of a role of cobalt deficiency in regulating microcystin-LR production.

This study enhances our understanding of cyanobacterial-metal interactions by demonstrating the importance of iron, cobalt and manganese for optimal growth. Interestingly, the absence of copper (a component of the thylakoid membrane and proteins in the electron-transport chain [14,17,70] did not appear to impact growth rate in *Microcystis aeruginosa*. This may indicate the substitution of copper-containing proteins (such as plastocyanin and Cu/Zn-SOD) with iron-containing proteins (such as cytochrome *c_6_* and Fe-SOD). A clear relationship was observed between iron internalisation and cobalt deficiency which is consistent with previous observations [12]. Intracellular iron was significantly higher in cobalt deficient cultures compared to the control and all other treatments. This may be due to the role of iron in the detoxification of ROS. Further, there was some evidence of cobalt-mediated microcystin-LR production, which requires further investigation.

## 4. Materials and Methods

### 4.1. Microcystis Culturing Conditions

Batch culture experiments were performed using the toxic *Microcystis aeruginosa* MASH01-AO5 (Australian National Algae Culture Collection, Hobart, Tasmania, Australia). Axenic cultures were maintained in MLA media [54] in an environmental chamber (Labec, HC-50 environmental chamber, Marrickville, Australia). Incubation was at 22 °C under 20–25 µmoles/m^2^/s light with a 14–10 h light-to-dark cycle throughout the long-term maintenance of the cultures as well as the duration of the experiment.

### 4.2. Culture Media

At the onset of the experiment, cells were subcultured into triplicate 700 mL sterile plastic culture flasks (Corning), which had previously been soaked overnight in an acid bath (10% HNO_3_ (*v*/*v*)) and rinsed repeatedly with Milli-Q water. Flasks contained 600 mL of filter sterilised MLA media, modified as described below. Table 1 summarises the concentrations of trace metals in unmodified MLA media (control treatment). 

In each experimental treatment one micronutrient was excluded from the growth media. Experimental treatments are summarised below. pH ranged between 7.4 and 7.7 in the growth media for all treatments.

(a) Control—filter sterilised MLA medium
(b) MLA media without CoCl_2_·6H_2_O
(c) MLA media without CuSO_4_·5H_2_O
(d) MLA media without FeCl_3_·6H_2_O
(e) MLA media without FeCl_3_·6H_2_O and Na_2_EDTA·2H_2_O
(f) MLA media without MnCl_2_·4H_2_O
(g) MLA media without Na_2_MoO_4_·2H_2_O

For each treatment an inoculum of *M. aeruginosa* was centrifuged at 3500× *g* for 10 min and the supernatant removed. The pellet was resuspended in the appropriate medium for the treatment. The centrifugation and resuspension steps were repeated to ensure there was no carry over of original media to the experimental cultures. A cell count was conducted on the washed, resuspended cells to calculate the volume of inoculum required to achieve an initial cell density of 10^4^ cell/mL. Once transferred a cell count was performed using a haemocytometer and cultures were maintained in the conditions outlined above. On day 31, 10^4^ cell/mL of *M. aeruginosa* were reinoculated into freshly prepared media to extend the exposure to the experimental conditions and assess the extent of luxury uptake and storage of micronutrients.

### 4.3. Sampling

Culture flasks were mixed by swirling and cell counts were conducted via optical density every 2–4 days (680 nm, 2 mL sample volume) (Varian Cary 50 Bio UV Spectrophotometer, Santa Clara, CA, USA). The relationship between *M. aeruginosa* cell count and absorbance at 680 nm was previously determined (R^2^ = 0.98). Manual cell counts were performed periodically using a haemocytometer to ensure manual cell counts were closely aligned with the optical density results. 

The nutrient/micronutrient composition of the culture media was sampled on day 0, 10, 20, 31 (before and after reinoculation), 40, 50 and 60 by filtering 25 mL of culture material through a 0.45 μm cellulose acetate syringe filter (Sartorius) prerinsed with 50 mL of 10% nitric acid followed by 100 mL milli-Q water. Samples were collected in acid washed 50 mL falcon tubes and refrigerated. Within 24 h of collection, samples were acidified with ultra-pure nitric acid to 0.2% *v*/*v*. Samples for intracellular microcystin-LR were taken from the inoculum on day 0 and days 20, 31, 50 and 60 from the experimental flasks. 10 mL of culture material from each replicate was filtered onto a Whatmans GF/C filter paper which was then stored in a −80 °C freezer. Intracellular iron was also sampled on day 0 from the inoculum and days 10, 20, 31, 40, 50 and 60 from the experimental flasks. Samples were prepared by transferring a volume of culture material corresponding to ~5 × 10^7^ cells (or ~0.97 pg dry weight) into acid washed, pre-weighed 50 mL falcon tubes. Transfers were performed immediately following a cell count. The culture material was centrifuged at 3500× *g* for 10 min to form a pellet. The supernatant was removed after ensuring the absence of cells by pipetting 1 mL of solution into a Sedgewick rafter counting chamber for observation using a light microscope (Olympus BX41, Tokyo, Japan). Samples were frozen at −20 °C. 

### 4.4. Solution Nutrient Determination

The concentration of nutrients (P, Co, Cu, Fe, Mn, Mo) in the filtered solution was analysed with a combination of inductively coupled atomic emission spectrometry (ICP-AES) (Varian 730 ES, Santa Clare, CA, USA) and inductively coupled plasma mass spectrometry (ICP-MS) (Agilent 7500 CE, Santa Clara, CA, USA). The spectrometer was operated according to the standard operating procedures outlined by the manufacturer. The instruments were calibrated using matrix-matched standards. At least 10% of samples were conducted in duplicate to ensure the precision of the analyses. To check for potential matrix interferences at least 10% of samples had spike recoveries performed.

### 4.5. Intracellular Iron Sample Preparation and Analysis

The falcon tubes were weighed to determine the volume of any overlying solution before freeze drying at 0.1 mbar and −80 °C until all liquid was sublimated from the samples. The dried pellet was submerged in 500 μL distilled nitric acid and microwaved at 80 °C with a 30 min holding time (CEM Mars 6, Matthews, SC, USA). Samples were diluted with 4.5 mL Milli-Q water and transferred to 5 mL acid washed vials for analysis via ICP-MS and ICP-AES. Analysis was performed using the instrument procedure outlined above.

### 4.6. Microcystin-LR Method

Filter papers were freeze dried (Martin Christ, alpha 2-4 LD plus, Osterode, Germany) at 0.1 mbar and −80 °C until all liquid was sublimated from the samples. Extraction was performed with 4 × 2 mL washes of 75% (*v*/*v*) aqueous methanol solution (methanol ≥ 99.9%, Sigma-Aldrich, Castle Hill, Australia) before sonication in a sonicator bath (Unisonics, Brookvale, Australia) for 15 min and centrifugation (Hettich Rotanta 460R, Tuttlingen, Germany) at 3500× *g* for 5 mins. The supernatant was transferred to a new 10mL centrifuge tube and dried using a dry block heater (Ratek, Boronia, Australia) at 40 °C under nitrogen. If any liquid remained it was removed by freeze drying. The sample was reconstituted in 1 mL of 10% methanol. A final filtration step was performed by transferring the sample to a microcentrifuge tube with 0.2 μM nylon filter (Corning Costar Spin-X, Sigma-Aldrich, Castle Hill, Australia) and centrifuging at 6500 for 5 min (Eppendorf, Centrifuge 5424 R, Hamburg, Germany). The samples were transferred into 2 mL amber vials (Supelco, Sigma-Aldrich, Castle Hill, Australia). 

The LC-MS analysis was performed on Thermo Scientific™ Q EXACTIVE™ high resolution mass-spectrometer (Waltham, MA, USA) equipped with an electrospray ionization source. The following source parameters were used in all experiments: a capillary temperature of 272 °C, a spray voltage of 3.5 kV, an auxiliary gas heater temperature of 425 °C, a sheath gas and an auxiliary gas flow rate of 54 and 14 (arbitrary units). The mass spectrometer was operated in negative ion mode scanning across the range of *m*/*z* 100–1150. Thermo Xcalibur software (version 3.0.63, Thermo Fisher Scientific, Inc., Waltham, MA, USA) was used for the data analysis.

Chromatographic separation was performed on a Thermo Scientific™ ACCELA™ UPLC system (Waltham, MA, USA). LC-MS analysis was performed by the method published by Turner et al. [80] which achieved recovery rates of between 94% and 79% in water. Separation was performed by an Acquity UPLC BEH Shield RP18 1.7 um, 2.1 mm × 50 mm column (Waters, Rydalmere, Australia) at temperature 40 °C. Mobile phases were A (ultrapure water + 0.025% formic acid) and B (acetonitrile + 0.025% formic acid). The LC-MS gradient and flow rate are shown in Table 2.

### 4.7. Cell Volume

Culture material was placed on a haemocytometer and photographed through a compound microscope (Olympus BX41, Tokyo, Japan). Images were processed using ImageJ software (Bethesda, MD, USA). Cells were measured when the treatment exhibited signs of growth limitation and were compared to the control. Treatments that did not exhibit growth limitation were measured at the completion of the experiment and compared to the control. 

### 4.8. Growth Rate

Specific growth rate was determined according to the following equation.

$$\mathrm{Growth}\;\mathrm{rate}\;(\mathrm{day}{\;}^{-1})=\frac{Ln\;C_{2}-Ln\;C_{1}}{\left(T_{2}-T_{1}\right)}$$

where *C*_1_ is the concentration of cells at time *T*_1_. *C*_2_ is the concentration of cells at time *T*_2_.

### 4.9. Data Analysis

A Kruskal–Wallis ANOVA on ranks with Dunn’s test was used to investigate differences in cell volume. Iron quota was analysed with a One-Way ANOVA and Tukey’s pairwise comparison was used to determine differences between treatments. Tests were performed using SigmaPlot 12.5 (San Jose, CA, USA) with a significance level of α = 0.05. The PERMANOVA (with Euclidian distances) was performed using the software PRIMER 7.0 (Auckland, New Zealand). Plots were created using the software R Version 1.2.1335 [81].

## Figures and Tables

**Table 1:** The composition of unmodified MLA algal growth media. Salts in bold text indicate those examined in this experiment.

Nutrient/Salt	Final Concentration (mg/L)
K_2_HPO_4_	34.80
NaNO_3_	170.00
NaHCO_3_	16.80
CaCl_2_	29.40
MgSO_4_·7H_2_O	49.10
H_3_BO_3_	2.40
CoCl_2_·6H_2_O	0.01
CuSO_4_·5H_2_O	0.01
FeCl_3_·6H_2_O	1.58
Na_2_EDTA·2H_2_O	4.56
MnCl_2_·4H_2_O	0.36
Na_2_MoO_4_·2H_2_O	0.006
ZnSO_4_·7H_2_O	0.022
Thiamine HCl	0.10
Biotin	5 × 10^−4^
Cyanocobalamin (B_12_)	5 × 10^−4^

**Table 2:** LC-MS gradient and flow rate for microcystin-LR analysis.

Time (min)	A%	B%	μL/min
0.00	98.0	2.0	600.0
0.50	75%	25.0	600.0
1.50	75%	25.0	600.0
3.00	60%	40.0	600.0
4.00	50%	50.0	600.0
4.10	5%	95.0	600.0
4.50	5%	95.0	600.0
5.00	98%	2.0	600.0
	100%	0.0	600.0

## Data Availability

Not applicable.

## Supplementary Materials

The following supporting information can be downloaded at: https://www.mdpi.com/article/10.3390/toxins14110812/s1, Table S1. Concentrations of micronutrients and P in each treatment (n = 3); Table S2. Recovery of Certified Reference Materials from ICP-AES analysis; Table S3. Recovery of Certified Reference Material from ICP-MS analysis.
