# Supplementary material for: The Influence of Micronutrient Trace Metals on Microcystis aeruginosa Growth and Toxin Production
Source: Toxins (Basel). 2022 Nov 21;14(11):812. doi: 10.3390/toxins14110812 (PMC9694995; doi:10.3390/toxins14110812)
Supplement: Supplementary file 1 [file toxins-14-00812-s001.zip › toxins-1991983-supplementary.pdf]

toxins

MDPI

Table S1. Concentrations of micronutrients and P in each treatment (n = 3).

Treatment	Day	Transfer	Co	Cu	Fe	Mn	Mo	P
			µg/L	*µg/L*	*µg/L*	*µg/L*	*µg/L*	*mg/L*
Control	0	1	2.5 ± 0.2	3.3 ± 0.1	302.6 ± 0.1	98.7 ± 0.3	2.9 ± 0	5.3 ± 0
-Co	0	1	<2	3.6 ± 0.4	298.5 ± 1.4	95.7 ± 0.5	3 ± 0.2	5.3 ± 0
-Cu	0	1	2.7 ± 0.3	<2	297.6 ± 2.2	97.6 ± 0.8	3 ± 0.2	5.3 ± 0
-Fe	0	1	2.9 ± 0.1	3.1 ± 0.2	1.1 ± 0	98.1 ± 0.3	2.8 ± 0.2	5.4 ± 0
-FeEDTA	0	1	2.9 ± 0	2.2 ± 0	4.3 ± 0.8	96.5 ± 1.6	2.8 ± 0.1	5.4 ± 0.1
-Mn	0	1	2.6 ± 0.1	4.9 ± 0.4	298.4 ± 1.9	0.8 ± 0.1	2.8 ± 0.1	5.3 ± 0.1
-Mo	0	1	3 ± 0.1	3.8 ± 0.1	304.9 ± 1.2	98.1 ± 0.7	<1 ± 0	5.4 ± 0
Control	10	1	2.8 ± 0.3	3.1 ± 0.1	286.2 ± 4.9	97.6 ± 0.6	2.5 ± 0	5.3 ± 0
-Co	10	1	<2	3.6 ± 0.3	285.9 ± 0.7	95.3 ± 0.3	2.5 ± 0	5.3 ± 0
-Cu	10	1	2.5 ± 0.2	1.5 ± 0	280.6 ± 7.8	96.3 ± 0.1	2.4 ± 0.2	5.3 ± 0
-Fe	10	1	2.6 ± 0	3.1 ± 0.1	<1	95.8 ± 0.6	2.4 ± 0.1	5.3 ± 0
-FeEDTA	10	1	2.5 ± 0	1.3 ± 0	<1	96.6 ± 0.7	2.5 ± 0.1	5.4 ± 0
-Mn	10	1	2.1 ± 0	4.9 ± 0.3	288.8 ± 3.4	0.4 ± 0.1	2.3 ± 0.2	5.2 ± 0
-Mo	10	1	2.3 ± 0	3.2 ± 0.1	285.4 ± 5.8	96.7 ± 0.6	<1	5.2 ± 0
Control	20	1	2.8 ± 0	2.5 ± 0	35.1 ± 8.2	67.3 ± 3.8	2.7 ± 0	3.7 ± 0.1
-Co	20	1	0 ± 0	3.4 ± 0.1	151.3 ± 24.8	100.5 ± 2.1	2.7 ± 0	4.9 ± 0.1
-Cu	20	1	2.7 ± 0	1.1 ± 0.2	54.6 ± 6.2	91.7 ± 1.6	2.7 ± 0	4.6 ± 0.1
-Fe	20	1	2.8 ± 0	3.3 ± 0	1.5 ± 0.2	102.6 ± 0.9	2.8 ± 0	5.4 ± 0
-Mn	20	1	2.8 ± 0.1	4.3 ± 0.2	87.4 ± 9.6	0.2 ± 0.1	2.7 ± 0.1	4.8 ± 0.1
-Mo	20	1	2.8 ± 0	2.8 ± 0.1	34.2 ± 8.9	77.9 ± 5.3	0 ± 0	4.2 ± 0.2
Control	31	1	2.8 ± 0	2.4 ± 0.1	26.5 ± 6.9	28.5 ± 5	2.7 ± 0	1.9 ± 0.1
-Co	31	1	0 ± 0	4 ± 0.1	125.6 ± 9.3	98.9 ± 0.8	2.7 ± 0	5 ± 0.1
-Cu	31	1	2.7 ± 0	1.3 ± 0.2	26.3 ± 3.8	35.4 ± 3.7	2.8 ± 0	2 ± 0.2
-Fe	31	1	2.9 ± 0	4.7 ± 0.6	2.1 ± 0	88 ± 2.9	2.9 ± 0	5.5 ± 0
-Mn	31	1	2.8 ± 0	4.5 ± 0.3	82.1 ± 13.9	0.1 ± 0	2.8 ± 0	3.8 ± 0.2
-Mo	31	1	2.8 ± 0	2.9 ± 0.1	22.3 ± 4.1	36 ± 4.3	0 ± 0	2.1 ± 0.1
Control	31	2	2.9 ± 0.1	3.6 ± 0.2	302.6 ± 1.3	98.1 ± 1	2.9 ± 0	5.1 ± 0
-Co	31	2	0 ± 0	3.2 ± 0.1	300.5 ± 0.8	97.2 ± 0.7	2.8 ± 0	5.3 ± 0
-Cu	31	2	2.9 ± 0	0.6 ± 0	316 ± 0.6	101.4 ± 0.5	2.9 ± 0	5.2 ± 0
-Fe	31	2	2.9 ± 0	3.4 ± 0	2.2 ± 0	101.6 ± 1.1	2.9 ± 0	5.3 ± 0
-Mn	31	2	2.8 ± 0	4.5 ± 0.1	304.9 ± 1.2	0.2 ± 0	2.8 ± 0	5.5 ± 0
-Mo	31	2	2.7 ± 0	5.1 ± 0.6	304.5 ± 1.2	95.5 ± 0.5	0 ± 0	5.2 ± 0
Control	40	2	2.7 ± 0	3 ± 0.1	50.9 ± 3.8	73.5 ± 0.7	2.8 ± 0	4.5 ± 0
-Co	40	2	<2	3 ± 0.1	264.2 ± 6.2	80.7 ± 0.6	2.7 ± 0	5.2 ± 0
-Cu	40	2	2.7 ± 0	0.5 ± 0	66.9 ± 12.6	87.9 ± 3.1	2.7 ± 0	4.6 ± 0.1
-Mn	40	2	2.7 ± 0	4.1 ± 0.1	101 ± 5.9	0.4 ± 0	2.7 ± 0	5.2 ± 0.1
-Mo	40	2	2.6 ± 0	4.4 ± 0.5	81.5 ± 18.6	81.2 ± 3.1	<0.01 ± 0	4.8 ± 0.1
Control	50	2	2.6 ± 0	2.5 ± 0.1	16.3 ± 3.9	40.5 ± 4.7	2.7 ± 0.1	2.6 ± 0.1
-Cu	50	2	2.8 ± 0	0.5 ± 0	33.5 ± 9.8	65.2 ± 7	2.9 ± 0	3.2 ± 0.2
-Mn	50	2	2.6 ± 0	3.6 ± 0.1	106.5 ± 21.3	0.1 ± 0	2.7 ± 0	5.1 ± 0
-Mo	50	2	2.6 ± 0	3.5 ± 0.4	13.7 ± 3.2	47.2 ± 3.6	<0.02 ± 0	2.7 ± 0.1
Control	60	2	2.5 ± 0	2.6 ± 0.1	14.7 ± 1.5	34.9 ± 2.1	2.7 ± 0	1.8 ± 0
-Cu	60	2	2.6 ± 0	0.7 ± 0	20 ± 2.7	42 ± 0.4	2.7 ± 0.1	1.9 ± 0.1
-Mn	60	2	2.6 ± 0.1	3.7 ± 0	104.1 ± 4.3	0.4 ± 0.1	2.7 ± 0.1	4.6 ± 0
-Mo	60	2	2.5 ± 0	4.1 ± 0.6	24.1 ± 1.5	26.7 ± 3.6	<0.02 ± 0	2 ± 0.1

Table S2. Recovery of Certified Reference Materials from ICP-AES analysis.

Sample	Co	Cu	Fe	Mn	Mo	P
TM-24.4 avg (n = 4)	µg/L	µg/L	µg/L	µg/L	µg/L	mg/L
	6.67	6.97	16.4	8.86	6.43	---
Certified Value	6.27 ± 0.57	6.31 ± 0.60	16.0 ± 2.3	8.24 ± 0.73	6.30 ± 0.56	---
Recovery (%)	106	110	102	108	102	---

Table S3. Recovery of Certified Reference Material from ICP-MS analysis.

Sample	Co	Cu	Fe	Mn	Mo
TM24.4 avg (n = 4)	6.49	6.41	15.6	8.64	6.21
TM-24.4 Certified	6.27 ± 0.57	6.31 ± 0.60	16.0 ± 2.3	8.24 ± 0.73	6.30 ± 0.56
Recovery (%)	104	102	98	105	98
TM64.3 avg (n = 4)	254	261	266	303	285
TMDA-64.3 Certified	250 ± 16	261 ± 18	298 ± 21	292 ± 17	286 ± 21
Recovery (%)	102	100	89	104	99
